# Neurodevelopmental disorder‐causing GRIN1 Y647S variant alters red blood cell physiology in mice

**DOI:** 10.14814/phy2.70759

**Published:** 2026-02-19

**Authors:** Sylvia C. Okafor, Wendy Horsfall, Peter S. B. Finnie, Caroline Holicka, Tao Wen, Behrooz Khatir, Melika Loriamini, Ali Salahpour, Kevin Golovin, Donald R. Branch, Graham R. Scott, Landon J. Edgar, Maggie L. Kalev‐Zylinska, Amy J. Ramsey

**Affiliations:** ^1^ Department of Pharmacology and Toxicology University of Toronto Toronto Ontario Canada; ^2^ Department of Biology McMaster University Hamilton Ontario Canada; ^3^ Department of Mechanical and Industrial Engineering University of Toronto Toronto Ontario Canada; ^4^ Canadian Blood Services Toronto Ontario Canada; ^5^ Department of Medicine University of Toronto Toronto Ontario Canada; ^6^ Department of Immunology University of Toronto Toronto Ontario Canada; ^7^ Department of Chemistry University of Toronto Toronto Ontario Canada; ^8^ Department of Molecular Medicine and Pathology University of Auckland Auckland New Zealand

**Keywords:** biomarker, GluN1, GRIN disorder, GRIN1‐related NDD, mouse model, non‐neuronal, pathogenic variant, red blood cells, NMDA receptor

## Abstract

GRIN Disorder is a rare neurodevelopmental disease caused by pathogenic variants in GRIN genes encoding subunits of the N‐methyl‐D‐aspartate receptor (NMDAR). GRIN Disorder presents with a wide spectrum of neurological symptoms and currently lacks effective therapeutics and clinically accessible biomarkers to stratify disease severity or monitor treatment response. While NMDARs are well‐studied in the central nervous system, they are also expressed in peripheral blood cells, including red blood cells (RBCs), where they modulate calcium signaling and cell function. Here we have used well‐established in vivo and ex vivo methods to investigate hematological (primarily RBC‐linked) phenotypes in transgenic mice carrying heterozygous Grin1 Y647S (Grin1^Y647S/+^) variant. We found that Grin1^Y647S/+^ mice had slightly increased RBC counts associated with increased erythropoiesis and normal erythropoietin levels. Functional assays revealed increased NMDAR‐mediated calcium influx in Grin1^Y647S/+^ RBCs, accompanied by reduced blood viscosity under flow conditions. Our findings provide the first genetic evidence that NMDAR gain‐of‐function leads to systemic changes in RBC physiology, which may contribute to core phenotypes of NMDAR‐related disorders. Results point to several RBC indices that reflect altered NMDAR function in Grin1^Y647S/+^ mice, providing foundational evidence for the development of peripheral blood biomarkers for patients with GRIN Disorder.

## Graphical abstract

Genetically‐modified mice with the Y647S substitution were used to study blood physiology and identify potential biomarkers for GRIN disorder patient stratification. Red blood cells from these mice have increased NMDA receptor mediated calcium influx, indicating a gain‐of‐function phenotype. The physiological impact of NMDAR gain‐of‐function included changes in blood viscosity and RBC number, providing new leads for biomarker discovery.

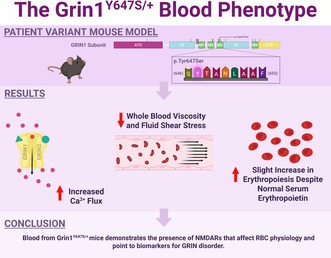

## 1 INTRODUCTION

GRIN Disorder is a group of neurodevelopmental diseases caused by pathogenic variants in *GRIN* genes, which encode the GluN subunits of the N‐methyl‐D‐aspartate receptor (NMDAR). The estimated incidence varies depending on the specific gene, but overall, the disorder is predicted to occur in one out of every 5208 births (Lemke, 2020). This debilitating condition primarily presents with neurological symptoms, including developmental delay, intellectual disability, epilepsy, autism, hypotonia, and visual impairment (Chen et al., 2017; Forrest et al., 1994; Lemke et al., 2016; Li et al., 1994; Mielnik et al., 2021; Mohn et al., 1999; Sullivan et al., 2024; Wesseling et al., 2014). Despite growing disease recognition, there are limited diagnostic and prognostic tools and no approved treatments.

Pathogenic single nucleotide variants in *GRIN1* and *GRIN2* genes underlie most GRIN Disorders, conferring gain‐ or loss‐of‐function of NMDAR ionotropic and metabotropic activity (Allen et al., 2013; Chen et al., 2017; Fry et al., 2018; Hamdan et al., 2011; Lemke et al., 2016; Ohba et al., 2015; Rossi et al., 2017; Sullivan et al., 2024; Venkatesan et al., 2024). Determining the functional impact of each variant is essential for treatment decisions, but this remains methodologically challenging, as direct assessment of brain tissue is not feasible in a clinical setting. There is thus an urgent need for accessible, peripheral blood biomarkers that can help stratify GRIN Disorders by functional impact, monitor disease activity over time, and assess treatment response. No such biomarkers currently exist, representing a major unmet clinical need.

While NMDARs are best known for their roles in synaptic plasticity and brain function—particularly learning and memory—they are also expressed in non‐neuronal tissues, including most blood cell types (Affaticati et al., 2011; Alim et al., 2020; Boldyrev et al., 2004; del Arroyo et al., 2019; Dickman et al., 2004; Hänggi et al., 2015; Hearn et al., 2020; Hearn et al., 2022; Hearn et al., 2023; Jeon et al., 2023; Kalev‐Zylinska et al., 2014; Kesarwani et al., 2023; Kopach et al., 2023; Kostanyan et al., 1997; Lombardi et al., 2001; Makhro et al., 2010; Makhro et al., 2013; Miglio et al., 2005; Orihara et al., 2010; Shang et al., 2010; Simma et al., 2014; Yuan et al., 2023). NMDAR‐mediated calcium flux influences many functional aspects of red blood cells (RBCs) (Hänggi et al., 2014; Hänggi et al., 2015; Hearn et al., 2020; Makhro et al., 2010; Makhro et al., 2013; Makhro et al., 2017), white cells (del Arroyo et al., 2019; Kopach et al., 2023; Shang et al., 2010), and platelets (Hearn et al., 2020; Hearn et al., 2022; Kalev‐Zylinska et al., 2014). In RBCs, the NMDAR is reported to regulate cell maturation, senescence, deformability, and oxygen carrying capacity (Hänggi et al., 2014; Hänggi et al., 2015; Hearn et al., 2020; Makhro et al., 2010; Makhro et al., 2013; Makhro et al., 2017). All GluN subunits except for GluN2B are expressed in RBCs (Hänggi et al., 2015; Makhro et al., 2010; Makhro et al., 2013). Early erythroid cells express primarily GluN2A and, as they mature, they are gradually replaced with GluN2C containing NMDARs (Hänggi et al., 2015). Additionally, the prevalence of NMDAR expression also decreases as they mature (Hänggi et al., 2015). Given what we know about the NMDAR and their proposed role in RBC physiology, we reasoned that pathogenic variants affecting NMDARs could alter blood cell properties in ways that could lead to biomarker development.

Transgenic mouse models with pathogenic *GRIN* variants have advanced our understanding of GRIN‐associated brain pathologies and identified new therapeutic targets (Allen et al., 2013; Benke et al., 2021; Burnashev & Szepetowski, 2015; Chen et al., 2017; Fry et al., 2018; Hearn et al., 2022; Iacobucci et al., 2022; Kolcheva et al., 2021; Mielnik et al., 2021; Milenkovic et al., 2014; Sullivan et al., 2018; Tang et al., 2020; Venkatesan et al., 2023; Xu et al., 2021; Zhang et al., 2021). However, the effects of these mutations on blood cell phenotypes have not been examined. In this study, we investigate peripheral blood phenotypes to search for accessible blood biomarkers using heterozygous Grin1^Y647S/+^ (Grin1^Y647S/+^) transgenic mice; modeled after a patient with GRIN1‐Related Neurodevelopmental Disorder (Kaniakova et al., 2012; Kolcheva et al., 2021; Sullivan et al., 2024; Venkatesan et al., 2024). We examined multiple hematologic parameters, including NMDAR‐mediated calcium flux in RBCs and whole blood hemorheological dynamics. Our results point to several RBC indices for further evaluation as peripheral blood biomarkers to assist in the care of patients with GRIN Disorder.

## 2 METHODS

### 2.1 Animals

All animal procedures were conducted in accordance with guidelines from the Canadian Council for Animal Care. The institutional approval was granted by the Faculty of Medicine and Pharmacy Animal Care Committee. Heterozygous Grin1^Y647S/+^ mice were created by CRISPR‐Cas9 endonuclease‐mediated transgenesis, maintained on a congenic C57Bl/6J background, and genotyped at weaning as described in Sullivan et al. (2024). Grin1^−/−^ knockdown mice were generated through targeted insertion of a neomycin cassette into intron 17 of the *GRIN1* gene (Mohn et al., 1999), maintained as F1 hybrids from the intercross of congenic C57Bl/6J and 129X1/SvJ heterozygotes as described in Milenkovic et al. (2014).

Mice were group‐housed with sex‐matched wildtype (WT) littermates in pathogen‐free microisolator cages at The Centre for Phenogenomics on a 12‐h light–dark cycle and ad libitum access to food, i.e. extruded pellet (TD.2918X; Inotiv), and water. Mice were euthanized via CO_2_ with cervical dislocation or isoflurane anesthesia with cervical dislocation. Every mouse was re‐genotyped after euthanasia using tail clippings to confirm the correct genotype.

### 2.2 Complete blood cell count

Saphenous vein blood of unfasted male and female mice that were 6 weeks of age. Blood was collected into K2 EDTA microvettes (Sarstedt). Complete blood counts (CBC) were measured within 2 h of collection on a Hemavet Hematology Analyzer (950FS) (Drew Scientific) by an experienced technician at The Centre for Phenogenomics.

### 2.3 Histology

Spleen, liver, and sternum were collected after euthanasia via CO_2_ and cervical dislocation of mice that were 8–10 weeks of age. Tissues were fixed in filtered 10% neutral buffered formalin (NBF) for 24–28 h at 4°C. Afterwards, the spleen and liver were rinsed in dH_2_O and transferred to 70% EtOH, while the sternum underwent decalcification in 20% EDTA for 5 days at 4°C on a rocker. EDTA was changed on the third day to ensure proper decalcification. After, the sternum was rinsed in dH_2_O and transferred to 70% EtOH. The tissues were embedded in paraffin wax. The spleen was embedded sagittally, the liver was embedded transversely, and the sternum was embedded longitudinally. 4 μm sections were cut and stained with either picrosirius red, H&E, or Prussian blue. H&E and Prussian blue slides were imaged with the AxioScan Slide Scanner (Axio Scan.Z1). They were then analyzed using QuPath (version 0.4.3). Slides stained with picrosirius red were imaged using a polarized light microscope to capture the birefringent collagen (Liu et al., 2021; Rittié, 2017). The images were then analyzed by using FIJI ImageJ.

The whole brain was collected from 3 to 5 month old male and female mice and placed into 10% filtered neutral buffered formalin after cervical dislocation under isoflurane anesthesia. The brain was fixed for 48 h at 4°C. Afterwards, it was rinsed in dH_2_O and transferred to 70% EtOH. The whole brain was cut along midline to produce 2 sagittal sections, then both sections were embedded sagittally at level 13 in paraffin wax. 5 μm serial sections were made by keeping sagittal sections 1.525 mm lateral of bregma. The sections were stained with Prussian blue and then imaged with an AxioScan Slide Scanner (Axio Scan.Z1). Images were analyzed using QuPath (version 0.4.3).

### 2.4 Picrosirius red FIJI ImageJ analysis

Thresholds were set for both collagen and background. This was used to measure mean gray scale value. The mean gray scale value of the collagen was subtracted from background to get the adjusted mean gray scale value which was then divided by the total area. This value was then multiplied by 100 to get percent collagen over the total area. The percent of collagen per μm^2^ was averaged between two serial sections.

### 2.5 QuPath analysis

The red pulp, white pulp, and marginal zone were annotated in QuPath (version 0.4.3). The mean marginal zone area was calculated by subtracting the total white pulp area from the total white pulp follicle area. The mean red pulp area was determined by subtracting total white pulp area from total spleen area. Prussian Blue stained slides were measured through training the pixel classifier tool to identify different variations of blue pixels that corresponded to hemosiderin staining. The classifier was then applied to the whole tissue. The percent of iron per μm^2^ was averaged between two serial sections. Analyzed images were double‐checked to make sure the classifier was measuring correctly.

### 2.6 Measuring basal erythroid populations in the bone marrow using spectral flow cytometry

Both femurs and tibias were collected after isoflurane anesthesia and cervical dislocation of mice that were 8–10 weeks of age. The top and bottom of the bone were cut off. Then the bone marrow was flushed out into a 40 μm mesh filter fitted onto a 50 mL tube. This was done using a 25 g syringe filled with ISCOVEs 1XDMEM supplemented with 10% FBS. Cells were then washed and used for investigating erythroid proliferation. Cells were incubated with Fixable Zombie NIR viability dye (BioLegend, 423,105). Afterwards, TruStain FcX™ (anti‐CD16/32, clone 93, BioLegend, 101,320) was applied. Cells were stained using the cell surface antibodies listed in Table 1.

**TABLE 1:** Markers used for erythroid differentiation profiling.

Marker (clone)	Fluorophore	Dilution	Concentration	Company and cat #
CD71 (OKT9)	AF647	1:500		BD; 566,725
TER119 (TER119)	FITC	1:250	0.5 mg/mL	BioLegend; 116,205
	DRAQ5	1:500	5 mM	BioLegend; 424,101
GRIN1 (N308/48)	PE (Streptavidin)	1:250 of Antibody 1:500 of Streptavidin	5 mM	BioLegend; 818,605 BioLegend; 405,204
CD45 (QA17A26)	PE/Cy7	1:500	0.2 mg/mL	BioLegend; 157,613

**FIGURE 1:**
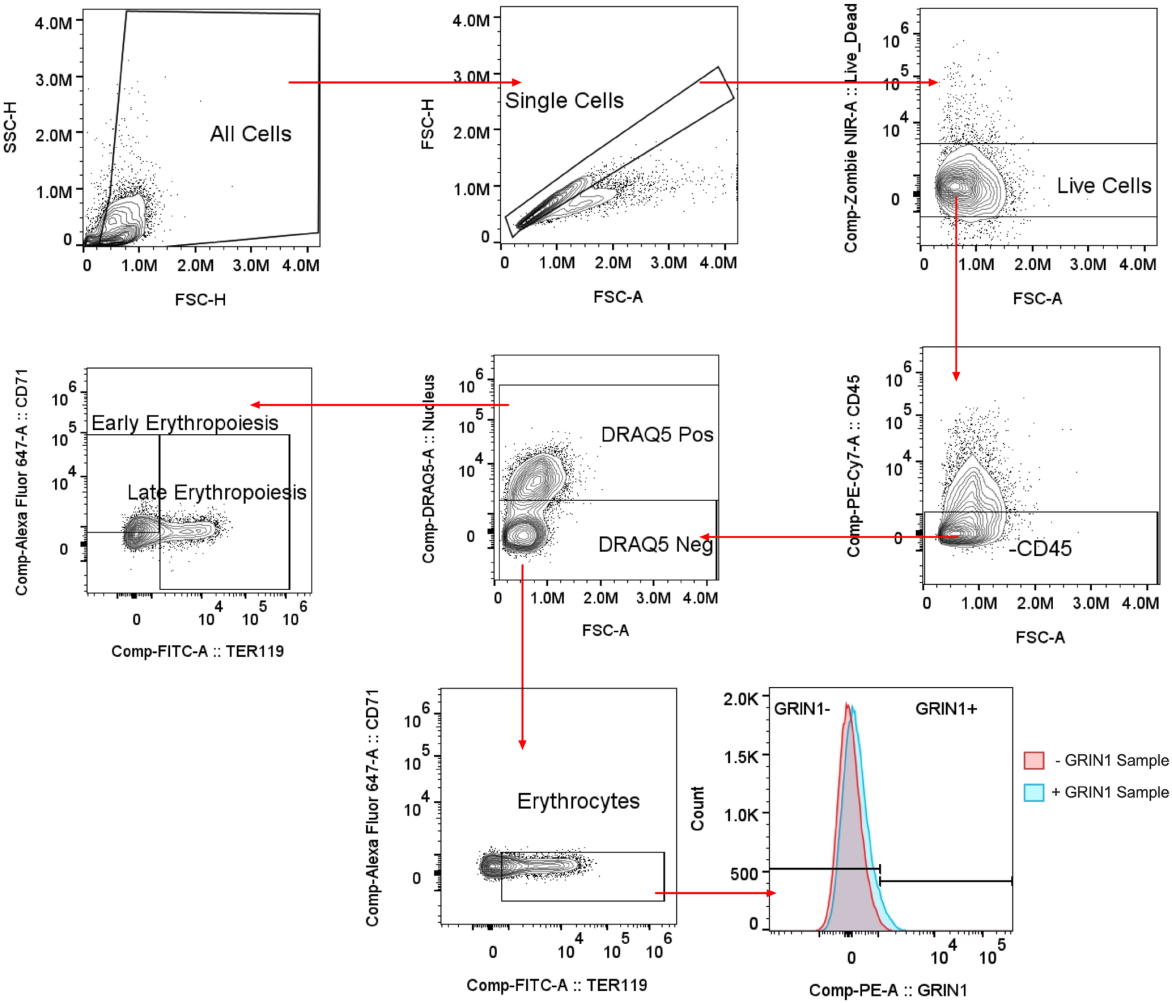
Gating strategy used for erythroid proliferation flow. GluN1 surface expression was determined using histograms as shown for RBCs.

PE streptavidin (BioLegend, 405,204) staining was completed afterwards. All incubations were conducted in the dark at room temperature (RT) for 30 min. Samples were analyzed using a Cytek Aurora Spectral flow cytometer equipped with 3 spatially separated lasers: 405 nm, 488 nm, and 640 nm. Unmixed data was analyzed using FlowJo (v10.10). Gating strategy is described in Figure 1. Early erythroid precursors (proerythroblasts) were defined as CD45^−^, DRAQ5^+^, CD71^high^, TER119^−^ cells, whereas late erythroid precursors (basophilic, polychromatic, and orthochromatic erythroblasts) were defined as CD45^−^, DRAQ5^+^, CD71^+/−^, TER119^−^ cells. Erythrocytes were identified as CD45^−^, DRAQ5^−^, CD71^−^, TER119^+^ (Boles et al., 2010; Kina et al., 2000; Shim et al., 2020; Tauzin et al., 2018).

### 2.7 Serum ELISA

Blood was collected via cardiac puncture from mice that were 8–10 weeks old and incubated at RT for 30 min. The blood was then centrifuged at 1500*g* for 13 min at 4°C. Serum was collected, aliquoted, and stored at −80°C until testing. A 1:4 dilution of serum was used to measure erythropoietin via Mouse Erythropoietin SimpleStep ELISA kit ab270893 (Abcam). Manufacturer's instructions were followed.

### 2.8 Live cell calcium imaging

Calcium imaging was done using slightly modified protocols described in several studies (Hänggi et al., 2014; Hänggi et al., 2015; Makhro et al., 2010; Makhro et al., 2013; Makhro et al., 2017). Blood was collected into heparinized microvettes from the saphenous vein of mice that were 9–14 weeks of age. 20 μL of blood was resuspended in ~600 mL of incubation medium (145 mM NaCl, 5 mM KCl, 0.15 mM MgCl_2_, 15 mM glucose, 2 mM CaCl_2_, and 10 mM Tris·HCl; pH 7.4). The samples were washed three times by centrifuging at 200*g* for 15 min without brakes and removing the supernatant. Samples were then resuspended in incubation medium supplemented with 4 μM Fluo‐8 am (Abcam, 1345980‐40‐6), which was diluted with DMSO. The samples were incubated for 2 h at RT in the dark on a vertical tube rotator. Afterwards, they were washed and resuspended with Tyrode solution (135 mM NaCl, 5.4 mM KCl, 10 mM glucose, 1 mM MgCl_2_, 10 mM HEPES, 2 mM CaCl_2_, and 0.1% BSA; pH 7.35). Samples were loaded onto an 8 chambered glass slide (BD Falcon, 08–774‐26) coated with Poly‐D‐lysine (Sigma, P7280‐5MG) and imaged on an Apotome Live Cell Microscope with 40X objective. Cell chambers received vehicle (Tyrode Solution) or drug at final concentrations of 200 μM NMDA (Sigma, M3262‐25MG) or 50 μM AP5 (Toronto Research Chemicals, A627550) plus 100 μM NMDA. Time‐lapsed videos were analyzed using FIJI ImageJ. 10 cells were blindly selected per animal using the ROI (region of interest) tool. The mean gray scale value of each ROI in the FITC channel was analyzed with multi‐measure. The mean gray scale value was subtracted from the background for each cell, then F/F0 was taken.

### 2.9 Whole blood hemorheology

Mice between the ages of 8–13 weeks were anesthetized with 3%–4% isoflurane carried in oxygen at a flow rate of 1 L/min, and blood was collected via cardiac puncture using a syringe flushed with 10% EDTA. 10% EDTA was added in a 1:9 ratio depending on the volume of blood collected (Baskurt et al., 2009). Low shear rates between ~10 and 110 s^−1^ are known to predominantly reflect RBC deformation and dynamic responses (Lanotte et al., 2016). At higher shear rates (~110–400 s^−1^), RBCs typically undergo morphological changes toward stomatocyte formation. To capture these dynamics, a Modular Compact Rheometer (Smartpave 102e) fitted with a 25.005 mm parallel plate (PP25, Anton Parr, part # 79044) was used to create viscosity curves in the Anton Parr Rheocompass software. The moving profile used the low viscosity setting. Gap height was set to 0.5 mm to assess blood viscosity and shear stress under flow conditions that mimic those in small veins and muscular arteries (vessel diameters ~0.1–10 mm) (Ostadfar, 2016). Measurements were taken at 37°C using a shear rate of 1–400 s^−1^. 400 measurement points were obtained and the interval between each measurement point was set to an initial of 10 s and a final of 1 s.

### 2.10 Hemox analysis

Using tail venipuncture, 10–20 μL of blood was collected into a heparinized glass capillary tube from mice that were 8–10 weeks of age. The blood was swiftly added to 5 mL of Hemox buffer on ice (50 mM HEPES, 10 mM EDTA, 100 mM NaCl; pH 7.4 at 37°C). The samples were then transported to McMaster University for analysis using a Hemox Analyzer (TCS Scientific). The Hemox Analyzer was calibrated according to manufacturer instructions. After calibration, the buffer containing the blood sample was added to the cuvette and 0.1% bovine serum albumin (0.1% of final concentration) and antifoaming agent (TCS Scientific; 0.2% final concentration) were added. The sample was then oxygenated at 37°C for 20 min. The gain (S1) was then set to a value between 2.4 and 2.6 and balance (S1/S2) to a value between 0.00 and 0.01. O_2_ dissociation curves and associated values of P_50_ (O_2_ partial pressure at 50% Hb saturation) were then measured at 37°C following recommendations of the manufacturer.

### 2.11 Statistical analysis

Statistical analysis was done in Prism v10.10 (GraphPad Software, Boston, USA). Outliers were determined with ROUT (Q = 1%). Statistical tests used to analyze the data are detailed in each figure legend. Statistical significance was achieved when *p values* were less than 0.05 (*p < 0.05; **p < 0.01; ***p < 0.001; ****p < 0.0001).

## 3 RESULTS

### 3.1 Grin1^Y647S^ ^/+^ mice have increased RBC count and erythropoiesis

Complete blood counts, performed on blood samples collected from the saphenous vein, revealed that Grin1^Y647S/+^ mice had subtle, yet statistically higher RBC parameters compared to WT. These include RBC count (*p* = 0.0067), hematocrit (*p* = 0.0105), and hemoglobin concentrations (*p* = 0.0087) (Table 2). A comparative analysis between sexes revealed a slight increase in RBC count for male Grin1^Y647S/+^ mice (*p* = 0.0282; Table 2) when compared to male WT mice. No other CBC measures displayed significant genotype or sex differences (Table 2).

**TABLE 2:** Complete blood cell (CBC) count data and sex comparisons. Data is reported as mean ± standard deviation. represent data from male mice and represents data from female mice. *Genotype statistics*: Two tailed unpaired *t*‐test. *n* = 24 WT animals and *n* = 22 Grin1^Y647S/+^ animals. *Sex Statistics*: Ordinary Two‐way ANOVA with Planned Multiple Comparisons. Alpha = 0.05. *n* = 13 male WT animals and *n* = 15 male Grin1^Y647S/+^ animals. *n* = 11 female WT animals and *n* = 10 female Grin1^Y647S/+^ animals.

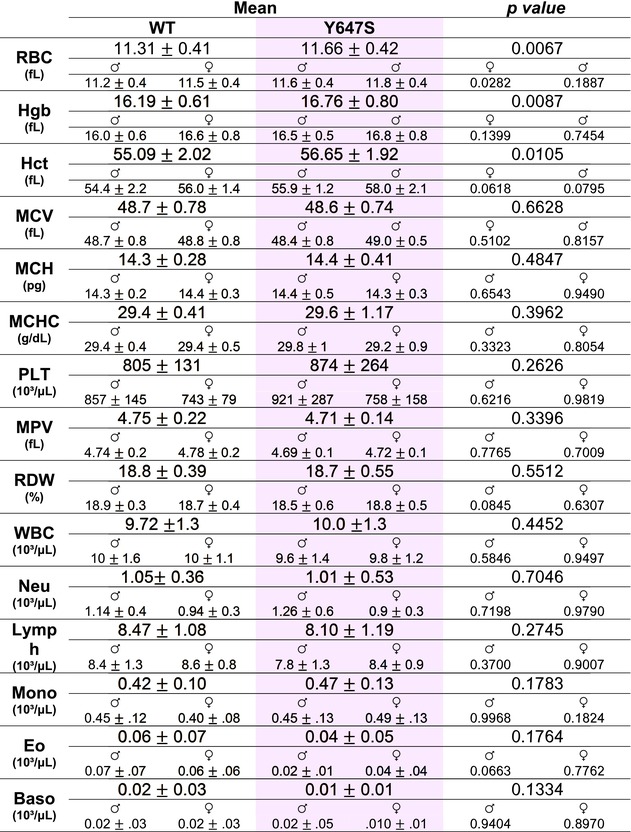

*Note*: The shaded color represents data from the patient varient mouse line.

Grin1^Y647S/+^ males and females both had lower bodyweights at 8–9 weeks of age relative to WT controls (*p* < 0.0001 and *p* = 0.0011, respectively; Table 3a). At 3–4 months, only the male mice remained significantly underweight (*p* < 0.0001; Table 3b). Grin1^Y647S/+^ *r*elative spleen weights were within the low‐normal range (Table 3).

**TABLE 3:** (a, b) Organ weight. (a) Body weight and organ weight at 8–9 weeks of age. *n* = 11 male WT animals and *n* = 11 male Grin1^Y647S/+^ animals. *n* = 11 female WT animals and *n* = 12 female Grin1^Y647S/+^ animals. (b) Body weight and organ weight at 3–5 months of age. *n* = 6 male WT animals and *n* = 6 male Grin1^Y647S/+^ animals. *n* = 5 female WT animals and *n* = 6 female Grin1^Y647S/+^ animals. *Statistics*: Ordinary Two‐way ANOVA with Planned Multiple Comparisons. Alpha = 0.05.

(a)		Body weight (g)		Splenic weight (mg)		Hepatic weight (mg)		Relative splenic weight (%)		Relative hepatic weight (%)	
		Mean	*p* value	Mean	*p* value	Mean	*p* value	Mean	*p* value	Mean	*p* value
	WT	25.5 ± 1.27	<0.0001	74.5 ± 8.78	0.5599	1506 ± 149	0.0030	0.292 ± 0.03	0.7315	5.90 ± 0.30	0.0925
	Y647S	22.3 ± 1.72		70.0 ± 10.9		1243 ± 187		0.304 ± 0.03		5.42 ± 0.70	
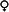	WT	20.0 ± 1.10	0.0011	79.7 ± 7.16	0.0001	1075 ± 122	0.0161	0.404 ± 0.04	0.0014	5.43 ± 0.35	0.1976
	Y647S	17.7 ± 1.60		58.1 ± 9.15		862 ± 132		0.339 ± 0.04		5.03 ± 0.46	

(b)		Body weight (g)		Splenic weight (mg)		Hepatic weight (mg)		Relative splenic weight (%)		Relative hepatic weight (%)	
		Mean	*p* value	Mean	*p* value	Mean	*p* value	Mean	*p* value	Mean	*p* value
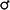	WT	28.8 ± 1.10	<0.0001	76.4 ± 5.81	0.0417	1479 ± 51.8	<0.0001	0.265 ± 0.02	0.9788	5.07 ± 0.18	0.4163
	Y647S	23.8 ± 1.32		62.3 ± 5.30		1150 ± 87.8		0.261 ± 0.02		4.82 ± 0.17	
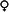	WT	20.3 ± 1.10	0.8268	76.9 ± 15.0	0.7184	987 ± 140	0.8182	0.378 ± 0.06	0.7761	4.84 ± 0.44	0.9879
	Y647S	19.9 ± 1.18		72.4 ± 11.6		954 ± 75.4		0.363 ± 0.04		4.80 ± 0.29	

**FIGURE 2:**
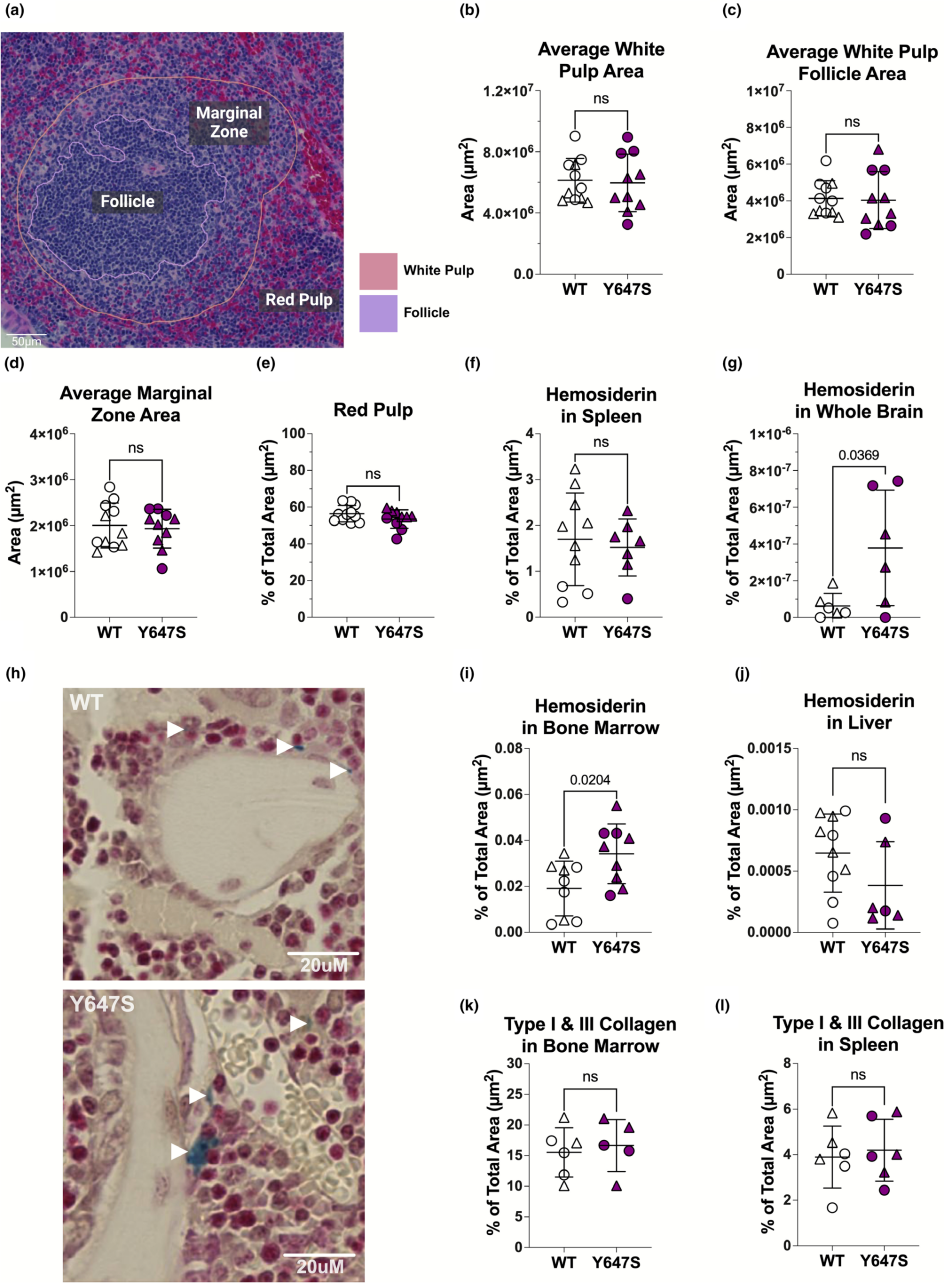
(a–l) Histology. Each point represents an individual WT animal (white) or Grin1^Y647S/+^ (purple) animal. Male mice are depicted as circles and female mice as triangles. (a) Spleen morphology was measured with QuPath and included the white pulp (highlighted in pink), white pulp follicle (highlighted in purple), marginal zone (outer region of the white pulp), and the red pulp area (remaining area). Image was captured with an AxioScan Slide Scanner (Axio Scan.Z1). Outlines were made using QuPath. (b) Average White Pulp Area (*p* = 0.8199). (c) Average White Pulp Follicle Area (*p* = 0.8657). (d) Average Marginal Zone Area (*p* = 0.7248). (e) Average Red Pulp Area (*p* = 0.1742). (f) Hemosiderin in spleen (*p* = 0.6884). (g) Hemosiderin in whole brain (*p* = 0.0369). (h) Hemosiderin in sternal bone marrow (*p* = 0.0204). (i) Representative Images of hemosiderin in the bone marrow. White arrows are pointing to hemosiderin deposits. (j) Hemosiderin in liver (*p* = 0.1464). (k) Type I and III collagen in sternal bone marrow (*p* = 0.6634). (l) Type I and III collagen in spleen (*p* = 0.7089). *Statistics*: Two tailed unpaired *t*‐test. Error bars represent SD. H&E Spleen Histology: *N* = 11 WT animals and *n* = 10 Grin1^Y647S /+^ animals. Prussian Blue Spleen Histology: *N* = 10 WT animals and *n* = 7 Grin1^Y647S /+^ animals. Prussian Blue Sternum Histology: *N* = 10 WT animals and *n* = 9 Grin1^Y647S/+^ animals. Prussian Blue Liver Histology: *N* = 10 WT animals and *n* = 6 Grin1^Y647S /+^ animals. Prussian Blue Brain Histology: *N* = 5 WT animals and *n* = 11 Grin1^Y647S /+^ animals. Picrosirius Red Spleen Histology: *N* = 5 WT animals and *n* = 5 Grin1^Y647S /+^ animals. Picrosirius Red Sternum Histology: *N* = 6 WT animals and *n* = 6 Grin1^Y647S /+^ animals.

Red pulp and white pulp organization appeared normal (Figure 2b–e). In the bone marrow we found no gross morphological differences. Although, Prussian blue staining of the bone marrow did reveal a slight increase in hemosiderin deposits in Grin1^Y647S/+^ mice (*p* = 0.0204; Figure 2i). Prussian blue staining also revealed a marked increase of hemosiderin deposits in the brains of Grin1^Y647S/+^ mice (*p* = 0.0369; Figure 2g). There were no remarkable findings in the liver (Figure 2j), nor were there any signs of reticulin or collagen fibrosis in the bone marrow or spleen (Figure 2k,l).

**FIGURE 3:**
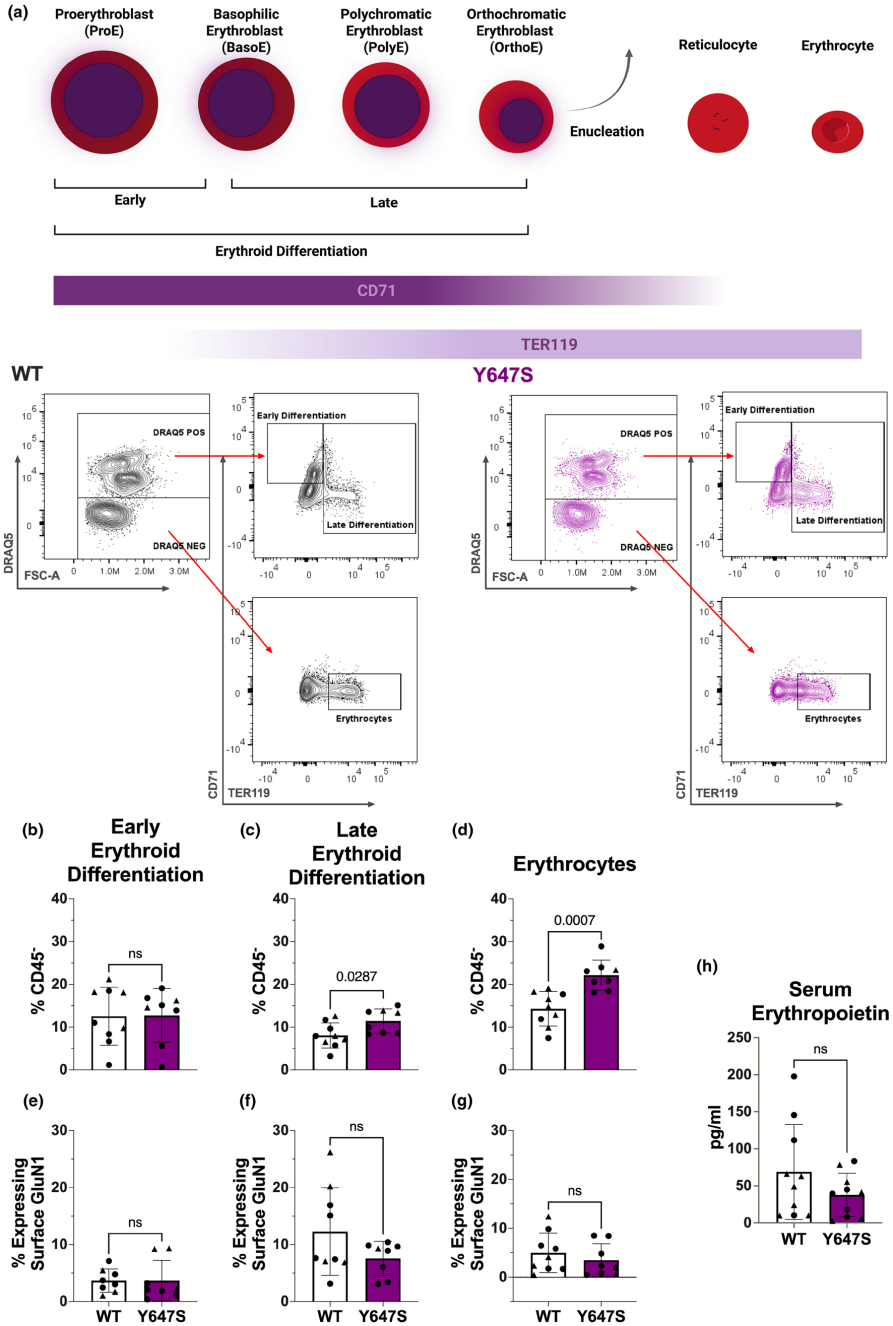
(a–h) Quantifying erythroid differentiation and serum EPO. (a) Simplified graphical diagram of the stages of erythroid maturation and the rationale behind our gating strategy. Made in Biorender 2025. (b) Bone marrow cells in the early stages of erythroid differentiation (CD45^−^, DRAQ5^+^, CD71^+^, TER119^−^) (*p* = 0.9509). (c) Bone marrow cells in the late stages of erythroid differentiation (CD45^−^, DRAQ5^+^, CD71^+/−^, TER119^+^) (*p* = 0.0287). (d) Erythrocytes found in the bone marrow (CD45^−^, DRAQ5^−^, CD71^−^, TER119^+^) (*p* = 0.0007). (e) GluN1 Surface Expression on bone marrow cells in the early stages of erythroid differentiation (*p* = 0.9986). (f) GluN1 Surface Expression on bone marrow cells in the late stages of erythroid differentiation (*p* = 0.1253). (g) GluN1 Surface Expression on erythrocytes in the bone marrow (*p* = 0.4204). (h) Serum Erythropoietin (EPO) concentrations determined with an ELISA (*p* = 0.1815). *Statistics*: Two tailed unpaired *t*‐test. Error bars represent SD. Erythroid differentiation: *N* = 9 WT animals and *n* = 8 Grin1^Y647S/+^ animals. Serum EPO ELISA: *N* = 10 WT animals and *n* = 10 Grin1^Y647S/+^ animals.

To investigate whether these RBC‐related differences were associated with more subtle alterations in erythropoiesis, we examined bone marrow samples isolated from femoral and tibial bones immediately after euthanasia. Spectral flow cytometry was used to quantify erythroid populations (Figure 3a).

Quantitative analysis revealed no significant difference in the percent of early erythroid precursors (proerythroblasts) in bone marrow aspirates from Grin1^Y647S/+^ mice compared to WT controls (12.75% ± 6.3% vs. 12.55% ± 6.8% in controls; *p* = 0.9509; Figure 3b). In contrast, late erythroid precursors (basophilic, polychromatic, and orthochromatic erythroblasts) were increased in Grin1^Y647S/+^ mice (11.44% ± 2.8% vs. 8.06% ± 2.9% in controls; *p* = 0.0287; Figure 3c). Bone marrow aspirates from Grin1^Y647S/+^mice also contained significantly more erythrocytes (22.16% ± 3.5% vs. 14.30% ± 4.0% in controls; *p* = 0.0007; Figure 3d).

Flow cytometric analysis confirmed cell surface expression of the GluN1 subunit—a requisite component of all NMDARs—on a subset of early and late erythroid precursors as well as on a subset of erythrocytes. Expression levels were comparable between genotypes across all erythroid populations (Figure 3e–g).

We next assessed if these erythroid changes were driven by altered levels of erythropoietin (EPO). Serum EPO concentrations, measured by ELISA, were comparable between WT and Grin1^Y647S/+^ mice (*p* = 0.1815; Figure 3h).

### 3.2 RBCs from Grin1^Y647S^ ^/+^ mice exhibit increased NMDAR–mediated calcium flux

To determine how Grin1^Y647S/+^ impacts NMDAR activity in RBCs, we measured calcium (Ca^2+^) flux in response to 200 μM NMDA, a selective NMDAR agonist. RBCs were immobilized on poly‐D‐lysine coated glass slides, loaded with the Ca^2+^‐sensitive dye, Fluo‐8 am, and imaged using live‐cell fluorescence microscopy.

**FIGURE 4:**
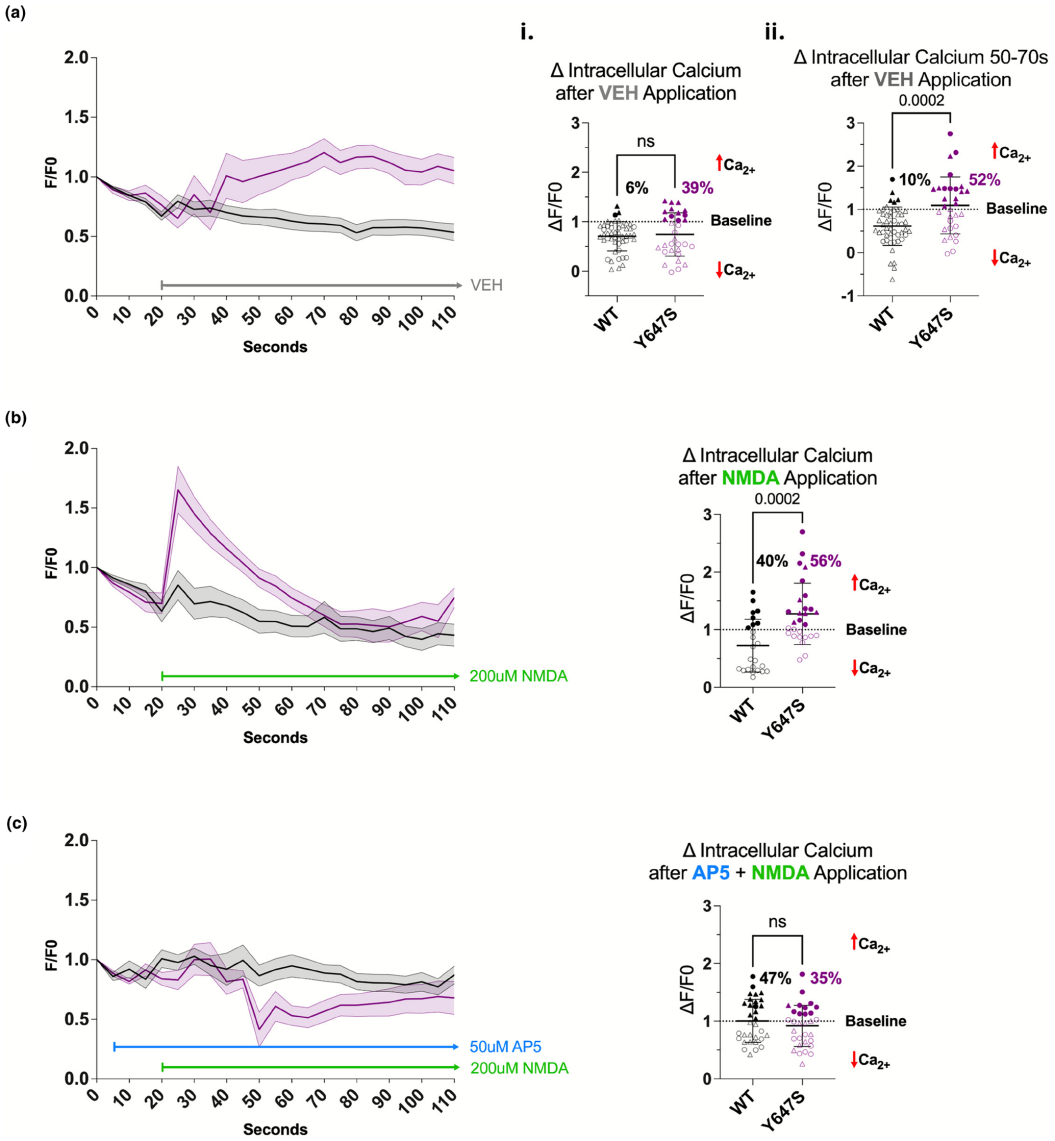
(a–c) RBC live cell calcium imaging. An open circle/triangle represents cells with calcium (Ca^2+^) efflux, while a closed circle/triangle represents cells with calcium influx. Cells for this study were incubated in Tyrode buffer supplemented with 2 mM CaCl_2_. Averages were taken between 25 and 35‐s. (ai) Average change from baseline after treatment with vehicle, i.e., Tyrode buffer (*p* = 0.6431). (aii) Average change from baseline 50 to 70‐s after treatment with vehicle (*p* = 0.0002). (b) Average change from baseline after treatment with 200 μM NMDA (*p* = 0.0002). (c) Average change from baseline after treatment with 50 μM AP5 (*p* = 0.9992) + 200 μM NMDA (*p* = 0.3555). *Statistics*: Two tailed unpaired t‐test. Error bars in line graphs represent SEM. Error bars in scatter plots represent SD. *n* = 24–49 WT RBCs from 7 animals and *n* = 28–31 Grin1^Y647S/+^ RBCs from 7 animals.

To rule out contributions from mechanosensitive Ca^2+^ channels, cells were first perfused with Tyrode buffer supplemented with 2 mM CaCl_2_ (vehicle). Vehicle treatment produced no significant change in Ca^2+^ flux in the first 25–35 s (*p* = 0.6431; Figure 4ai). However, 50–70 s after the application of vehicle, Ca^2+^ flux increased in Grin1^Y647S/+^ when compared to WT (*p* = 0.0002; Figure 4aii). Due to this, we limited our analysis to 10 s after treatment.

Application of NMDA (200 μM) induced Ca^2+^ influx in approximately half of all measured RBCs from both genotypes (Figure 4c). However, Grin1^Y647S/+^ RBCs showed significantly greater Ca^2+^ flux when compared to WT (*p* = 0.0002; Figure 4d). Pre‐treatment with the competitive NMDAR antagonist AP5 (50 μM) abolished the Ca^2+^ response (*p* = 0.3555; Figure 4e), confirming that calcium flux was indeed primarily NMDAR–mediated. In all, the Grin1^Y647S/+^ variant enhanced NMDAR–mediated Ca^2+^ entry in RBCs, indicating a gain‐of‐function for calcium ion flux.

### 3.3 Blood viscosity and fluid shear stress are reduced in Grin1^Y647S^ ^/+^ mice

**FIGURE 5:**
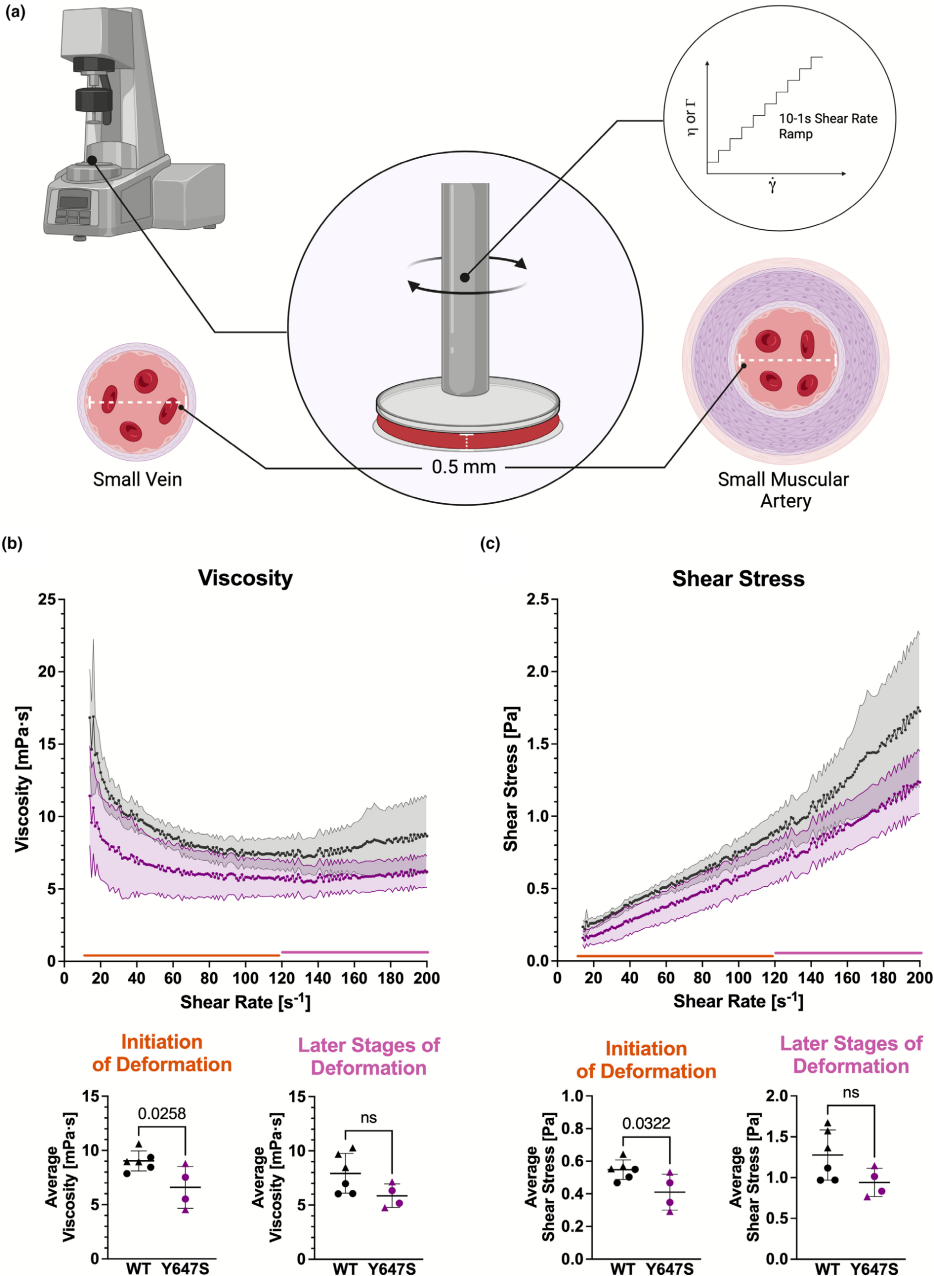
(a–c) Grin1^Y647S/+^ hemorheology. Measurements were taken at 37°C using a parallel plate rheometer and a 25 mm plate. (a) Graphic representation of the viscosity protocol used to model blood flow through small muscular arteries and veins. Made in Biorender 2025. The gap width was set to 0.5 mm. Viscosity (η). Shear Stress (τ). Shear rate ($\dot{\gamma }$). An ascending shear rate ramp was used, and 400 measurement points were taken. (b) Average viscosity obtained using the described protocol. Measurements taken at the initiation of RBC deformation (from 14 to 119 s^−1^, shown in green) (*p* = 0.0258) and in the latter stages of deformation (from 120 to 200 s^−1^, shown in pink) (*p* = 0.0800). (c) Average shear stress obtained using the described protocol. Measurements taken at the initiation of RBC deformation (from 14 to 119 s^−1^) (*p* = 0.0322) and in the latter stages of deformation (from 120 to 200 s^−1^) (*p* = 0.0831). *Statistics*: Two tailed unpaired *t*‐test. Error bars in line graphs represent SEM. Error bars in scatter plots represent SD. *n* = 6 WT animals and *n* = 4 Grin1^Y647S/+^ animals.

We then investigated the hemorheological properties of whole blood collected from mutant and WT mice, since RBC Ca^2+^ dynamics are important for deformation (Bogdanova et al., 2013; Fermo et al., 2017; Makhro et al., 2013) and subsequently hemorheology (Lanotte et al., 2016; Reichel et al., 2019). We employed a parallel plate rheometer using a protocol we designed to assess blood viscosity and shear stress under flow conditions that mimic those in small veins and muscular arteries (vessel diameters ~0.1–10 mm) (Ostadfar, 2016). To capture these dynamics, we applied an ascending shear rate ramp protocol and maintained a gap height of 0.5 mm to reflect the geometric constraints of small vessels (Figure 5a). In this paradigm we found that whole blood from the Grin1^Y647S/+^ was less viscous (*p* = 0.0258; Figure 5b) and had less shear stress (*p* = 0.0322; Figure 5c) during the early phases of deformation (14–120 s^−1^) when compared to WT.

**FIGURE 6:**
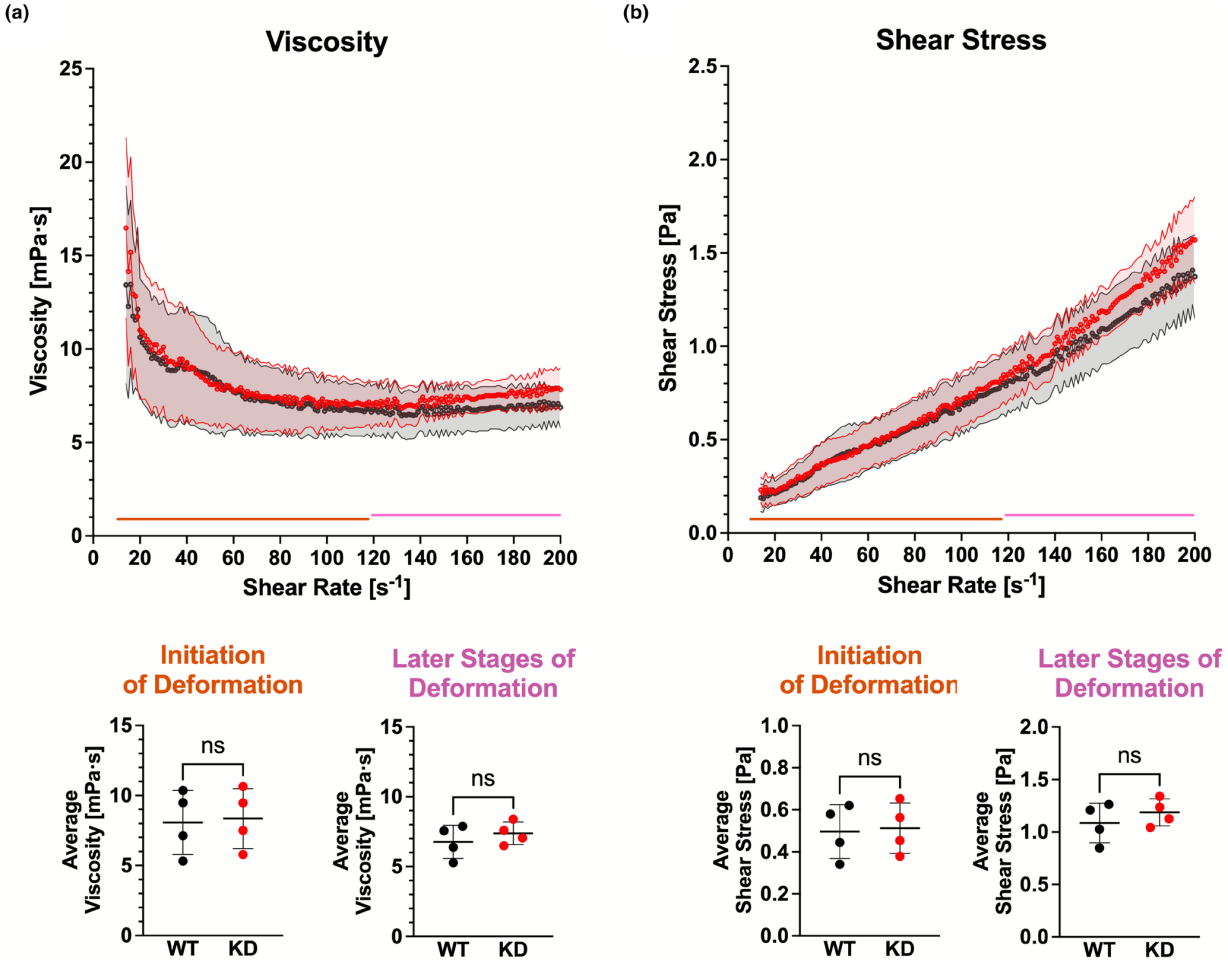
(a, b) Grin1^−/−^ KD hemorheology. (a) Average viscosity obtained using the described protocol. Measurements taken at the initiation of RBC deformation (from 14 to 119 s^−1^) (*p* = 0.8651) and in the latter stages of deformation (from 120 to 200 s^−1^) (*p* = 0.4323). (b) Average shear stress obtained using the described protocol. Measurements taken at the initiation of RBC deformation (from 14 to 119 s^−1^) (*p* = 0.8655) and in the latter stages of deformation (from 120 to 200 s^−1^) (*p* = 0.4134). *Statistics*: Two tailed unpaired t‐test. Error bars in line graphs represent SEM. Error bars in scatter plots represent SD. *n* = 4 WT animals and *n* = 4 Grin1^−/−^ KD animals.

To determine if the NMDAR could directly impact hemorheology, we repeated this experiment using whole blood from Grin1^−/−^ knockdown (KD) mice. These mice have a 90% reduction in Grin1 mRNA and protein (Mielnik et al., 2021; Mohn et al., 1999). Intriguingly, there was no significant difference between Grin1^−/−^ KD mice and their WT littermates (Figure 6a,b). Thus, while Grin1^Y647S/+^ caused significant alterations in hemorheology, the Grin1^−/−^ KD did not produce any remarkable hemorheological changes.

### 3.4 Grin1^Y647S^ ^/+^ RBCs display normal oxygen‐carrying function

**FIGURE 7:**
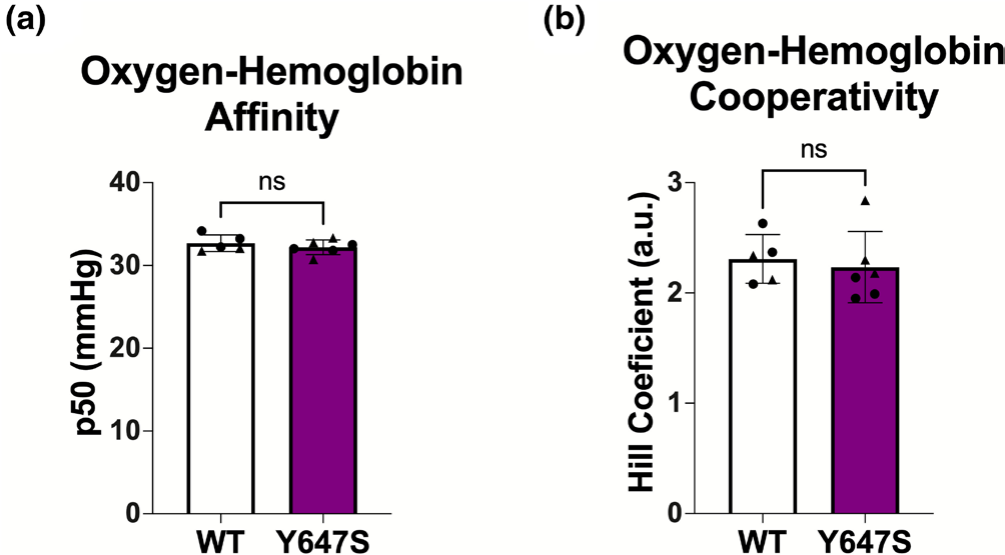
(a, b) Measuring hemoglobin function. a.u. = arbitrary units. (a) Oxygen‐hemoglobin cooperativity (*p* = 0.6730). (b) Oxygen‐hemoglobin affinity (*p* = 0.4083). *Statistics*: Two tailed unpaired *t*‐test. *n* = 5–6 WT animals and *n* = 6 Grin1^Y647S/+^ animals.

Homeostatic hemorheology can impact oxygen‐hemoglobin dissociation (Connes et al., 2016; Lanotte et al., 2016). We therefore examined oxygen‐hemoglobin dissociation using a Hemox Analyzer. We found that oxygen‐hemoglobin cooperativity, as reflected by the Hill coefficient describing the shape of the oxygen equilibrium curve, was unchanged in Grin1^Y647S/+^ samples compared to WT (*p* = 0.6730; Figure 7a). Oxygen‐hemoglobin affinity, detected as the partial pressure of O_2_ at 50% hemoglobin saturation (p50), was also unchanged (*p* = 0.4083; Figure 7b).

## 4 DISCUSSION

Our study has uncovered several lines of evidence indicating altered RBC properties in Grin1^Y647S/+^ mice. We found that Grin1^Y647S/+^ mice had increased numbers of late erythroblasts and mature RBCs, increased RBC Ca^2+^ flux, reduced whole blood viscosity and shear stress, and increased hemosiderin deposits in the bone marrow and brain. Remarkably, we found no difference in serum EPO concentration despite an increase in RBC count. Furthermore, our flow cytometric analysis of the bone marrow supports existing lines of evidence that suggest NMDAR–mediated Ca^2+^ signaling contributes to RBC differentiation (Hänggi et al., 2015). Lastly, our findings highlight new roles for NMDARs in the modulation of whole blood hemorheology. In all, these findings provide new avenues for the development of peripheral blood biomarkers for patients with GRIN1 disorder.

Functional characterization and classification of the Grin1^Y647S/+^ variant has been challenging. Several studies report mixed effects in cell culture systems and within the brain (Functional Variants Center Database, 2017‐2023; Kaniakova et al., 2012; Kolcheva et al., 2021; Lemke et al., 2016; Sullivan et al., 2024; Venkatesan et al., 2024). For example, in HEK cells, the Grin1^Y647S/+^ variant has a mixed phenotype, characterized by increased charge transfer yet reduced surface expression (Kaniakova et al., 2012; Kolcheva et al., 2021; Sullivan et al., 2024). A study done with xenopus oocytes describes loss‐of‐function due to reduced maximal agonist inducible currents (Lemke et al., 2016). While another describes increased sensitivity to L‐glutamate and glycine (Functional Variants Center Database, 2017‐2023; Sullivan et al., 2024). Patch recordings from isolated murine NMDARs were found to produce decreased currents (Venkatesan et al., 2024). While whole cell recordings revealed a marked increase in integrated and circuit‐level NMDAR signaling (Venkatesan et al., 2024). Due to these conflicting observations, it was important to, first, determine how the variant affected NMDARs in RBCs. In this study, we revealed that RBCs from the Grin1^Y647S/+^ mice displayed gain‐of‐function characteristics given the increased amount of Ca^2+^ flux seen in Ca^2+^ imaging.

Our hemorheological assays revealed reduced whole blood viscosity and shear stress in Grin1^Y647S/+^ mice. Although hemorheology was altered for Grin1^Y647S/+^ mice, we did not observe any changes using Grin1^−/−^ KD mice. This suggests that excessive NMDAR activation can influence hemorheology, but under normal physiological conditions NMDARs may not have an active role in hemorheology. This is notable as a recent study found no discernable difference between healthy RBCs that were given NMDAR agonist, homocysteine, or antagonist, memantine (Reinhart et al., 2025). Though, it is important to note that unlike their study, our assay used EDTA as an anticoagulant since heparin has a greater influence on hemorheological parameters (Baskurt et al., 2009; Baskurt & Meiselman, 2003). Since EDTA is a Ca^2+^ chelator our data also suggests that the Grin1^Y647S/+^ variant might also influence other aspects of hemorheology that are not Ca^2+^ dependent. Normal hemorheological function is largely maintained by RBC deformability (Bogdanova et al., 2013; Hertz et al., 2017; Kim et al., 2015; Petkova‐Kirova et al., 2024; Richardson & Swietach, 2016). Perhaps examining RBC deformation could yield more potential biomarkers for clinical use. Other fluid shear stress‐mediated processes (leukocyte migration/function/Ca^2+^ release, and platelet formation/clotting (Murphey et al., 2022; Son et al., 2021)) could likewise reveal additional changes that could be used as potential blood biomarkers.

In addition to hemorheology, we also investigated oxygen‐hemoglobin dissociation as hemorheology and RBC deformation are important for oxygen‐hemoglobin dissociation (Richardson et al., 2020; Richardson & Swietach, 2016). Despite atypical hemorheology, we found no change in basal Grin1^Y647S/+^ oxygen‐hemoglobin dissociation curves. The lack of difference was surprising given that NMDAR‐mediated Ca^2+^ signaling was found to facilitate oxygen‐hemoglobin binding in RBCs by modulating oxygen carrying capacity (Makhro et al., 2010). However, our study did not examine the effects of NMDAR activation/inhibition on oxygen‐hemoglobin dissociation, nor did we examine how nitric oxide‐hemoglobin binding might be affected. Future studies investigating these properties could be beneficial. In addition, in vivo studies are needed to determine whether tissue from Grin1^Y647S/+^ mice is properly oxygenated, which would have implications for multiple aspects of brain function including seizure susceptibility (Ingram et al., 2014).

Lastly, we discovered that NMDAR gain‐of‐function upregulates erythroid differentiation despite normal EPO concentrations. Our histological findings in the spleen and liver argue against RBC sequestration and turnover. Thus, Grin1^Y647S/+^ RBCs likely have a normal lifespan which suggests that NMDAR gain‐of‐function alters erythropoiesis. This notion is further supported by Hänggi et al. (2015), who discovered that NMDAR–mediated Ca^2+^ signaling can modulate Ca^2+^ homeostasis in late erythroid precursors (Hänggi et al., 2015). Moreover, EPO promotes erythropoiesis via the activation of JAK2, STAT3, and STAT5 (Ayele et al., 2022; Hu et al., 2021; Levine et al., 2007). Interestingly, there is evidence of crosstalk between the JAK2/STAT3 pathway and NMDARs. Several studies have demonstrated that both JAK2 and STAT3 can modulate NMDAR‐dependent long‐term depression in the hippocampus (McGregor et al., 2017; Nicolas et al., 2012). Furthermore, activated NMDARs can act on the JAK2/STAT3 pathway via a Ca^2+^ dependent signaling cascade that phosphorylates JAK2 and STAT3 (McGregor et al., 2017; Nicolas et al., 2012). These known links between NMDAR activation and JAK2/STAT3 signaling could also explain the mild increase in erythroid differentiation. Future studies investigating NMDAR dysfunction and its impact on EPO and the JAK2/STAT3 pathway are warranted.

To conclude, hematological phenotyping of Grin1^Y647S/+^ mice provides strong genetic evidence that corroborates previous pharmacological studies and demonstrates that NMDARs have physiological roles in erythroid development and physiology. This study is the first genetic evidence that blood can be used to directly study NMDAR function in GRIN1 disorder. Testing patient blood samples using identical techniques is the next step to determine whether these assays could be used as robust biomarkers for clinical and diagnostic use.

## AUTHOR CONTRIBUTIONS

S.C.O., P.S.B.F., M.L.K.‐Z., L.J.E., and A.J.R. contributed to the conception and design of the study. S.C.O., P.S.B.F., M.L.K.‐Z., G.S., L.J.E., and A.J.R. contributed to drafting the text. S.C.O., P.S.B.F., M.L.K‐Z., L.J.E., and A.J.R. contributed to preparing the figures. S.C.O., W.H., C.H., T.W., B.K., M.L., D.R.B., L.J.E., and M.L.K‐Z. contributed to the acquisition and analysis of data.

## FUNDING INFORMATION

Simons Foundation Autism Research Initiative (SFARI): Amy J Ramsey; CureGrin Foundation: Maggie L Kalev‐Zylinska, Amy J Ramsey; Canadian Institutes of Health Research (CIHR): Amy J Ramsey, FRN169153.

## CONFLICT OF INTEREST STATEMENT

The authors declare no competing financial interests.

## Data Availability

The data that support the findings of this study are available from the corresponding author upon reasonable request.
